# Hydroxysafflor yellow A: a natural pigment with potential anticancer therapeutic effect

**DOI:** 10.3389/fphar.2024.1495393

**Published:** 2025-01-14

**Authors:** Yuhan Wang, Junsha An, Jianbo Zhou, Liming Chang, Quan Zhang, Fu Peng

**Affiliations:** ^1^ Key Laboratory of Drug-Targeting and Drug Delivery System of the Education Ministry, Sichuan Engineering Laboratory for Plant-Sourced Drug and Sichuan Research Center for Drug Precision Industrial Technology, West China School of Pharmacy, Department of Pharmacy, West China Hospital, Chengdu, China; ^2^ Sichuan Province College Key Laboratory of Structure-Specific Small Molecule Drugs, School of Pharmacy, Chengdu Medical College, Institute of Materia Medica, Chengdu, China; ^3^ Development and Regeneration Key Lab of Sichuan Province, Department of Pathology, Chengdu Medical College, Chengdu, China; ^4^ Development and Regeneration Key Lab of Sichuan Province, Department of Anatomy and Histology and Embryology, Chengdu Medical College, Chengdu, China

**Keywords:** hydroxysafflor yellow A, pharmacological effects, anticancer effect, pharmacokinetic progress, safety

## Abstract

Hydroxysafflor yellow A (HSYA), a natural pigment with a chalcone structure extracted from *Carthamus tinctorius L.* (Safflower), has been widely proven to have good efficacy on cardiovascular diseases, atherosclerosis, cancer, and diabetes. However, no study has reported on the anticancer mechanisms of Hydroxysafflor yellow A (HSYA), a principal bioactive compound in safflower. This review discusses recent developments in the physicochemical properties and sources, pharmacological effects and mechanisms, pharmacokinetic progress, and safety of HSYA, focusing on the involvement of HSYA in the regulation of related pathways and mechanisms of apoptosis, autophagy, and the tumor immune microenvironment in a variety of cancers. This can serve as a theoretical basis for further research and development of HSYA, with insights into the mechanisms of anticancer signaling pathways.

## 1 Introduction

Cancer is a malignant tumor resulting from abnormal cell proliferation, malignant infiltration, and growth. According to the GLOBOCAN Statistical Report, cancer was the second leading cause of mortality worldwide from 2018 to 2020. Notably, it is the leading cause of death among individuals under the age of 70 in nearly 60% of countries (Sung et al., 2021; Ugai et al., 2022). The global incidence of new cancer cases in 2020 is estimated to reach approximately 19.3 million, with an associated mortality rate of 10 million. The most common types of cancer based on age-standardized mortality rates in all countries and regions in 2020 were lung cancer (60 countries), breast cancer (52 countries), prostate cancer (32 countries), and cervical cancer (28 countries). Breast cancer was the leading type of cancer in terms of age-standardized incidence rate in 109 countries in 2020 (Siegel et al., 2023). According to data from the World Health Organization, the number of new cancer cases worldwide is projected to exceed 28 million by 2040 (Williams et al., 2022), indicating that one-fifth of the global population will be affected by cancer. Consequently, cancer prevention has become a critical public health issue in the 21st century (Rumgay et al., 2022), and the development of new cancer therapies and targets is of urgent importance.

*Carthamus tinctorius L.* (Safflower), is an annual or biennial herbaceous plant belonging to the Compositae family (Zhang et al., 2016). It thrives in warm and dry climates, and is resistant to cold conditions. Native to Central Asia, safflower is now widely cultivated in China, Japan, and North Korea (Ao et al., 2018). In traditional Chinese medicine, safflower typically refers to the dried flowers of the plant, which were first documented in Zhang Zhong Jing’s ‘Synopsis of the Golden Chamber’ for the treatment of gynecological disorders (Adamska and Biernacka, 2021). In modern Chinese medicine, safflower is used primarily to promote blood circulation, reduce blood stasis, and alleviate discomfort. Hydroxysafflor yellow A (HSYA), a chalcone glycoside with good water solubility, is the principal bioactive compound found in safflower. Due to its low abundance and efficacy, HSYA is a key quality control standard in the Chinese Pharmacopoeia. Currently, research on HSYA has mainly focused on its effects on cardiovascular diseases (Bai et al., 2020). Safflower yellow (SY) injection, an NMPA-approved drug for treating angina pectoris and cerebral infarction, contains up to 90% HSYA as the main ingredient (each 50 mg of SY injection contained 45 mg of HSYA) (Li et al., 2012). HSYA also exhibits various other biological activities, including neuroprotective, anticoagulant and anti-tumor activities, and potential benefits against hepatic fibrosis, pulmonary arterial hypertension, diabetes, and vascular dementia (Zhang et al., 2021).

**FIGURE 1:**
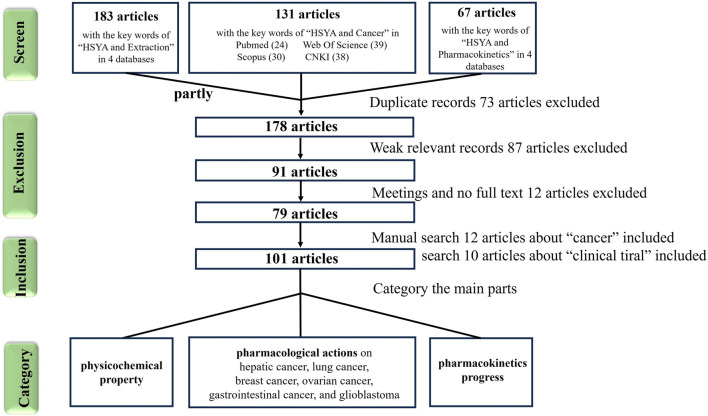
The screen flowchart of cancer research articles related to hydroxysafflor yellow A in databases.

In the past 2 years, literature has reviewed and discussed the mechanisms of HSYA in treating cardiovascular and cerebrovascular diseases (Bai et al., 2020), atherosclerosis (Xue et al., 2021), and diabetes (Zhang et al., 2021). However, the its anticancer effects have not been comprehensively summarized, and the its mechanisms involved in treating various cancers (pan-cancer) remain unclear. Therefore, this review collected articles containing the keywords “cancer” and “HSYA” from four databases: “PubMed,” “Web of Science (WOS),” “Scopus” and “CNKI.” Relevant original articles were categorized and summarized (Figure 1) to consolidate the recent research on the anticancer effects of HSYA and discuss its underlying molecular mechanisms.

## 2 The physicochemical properties and resources of HSYA

**FIGURE 2:**
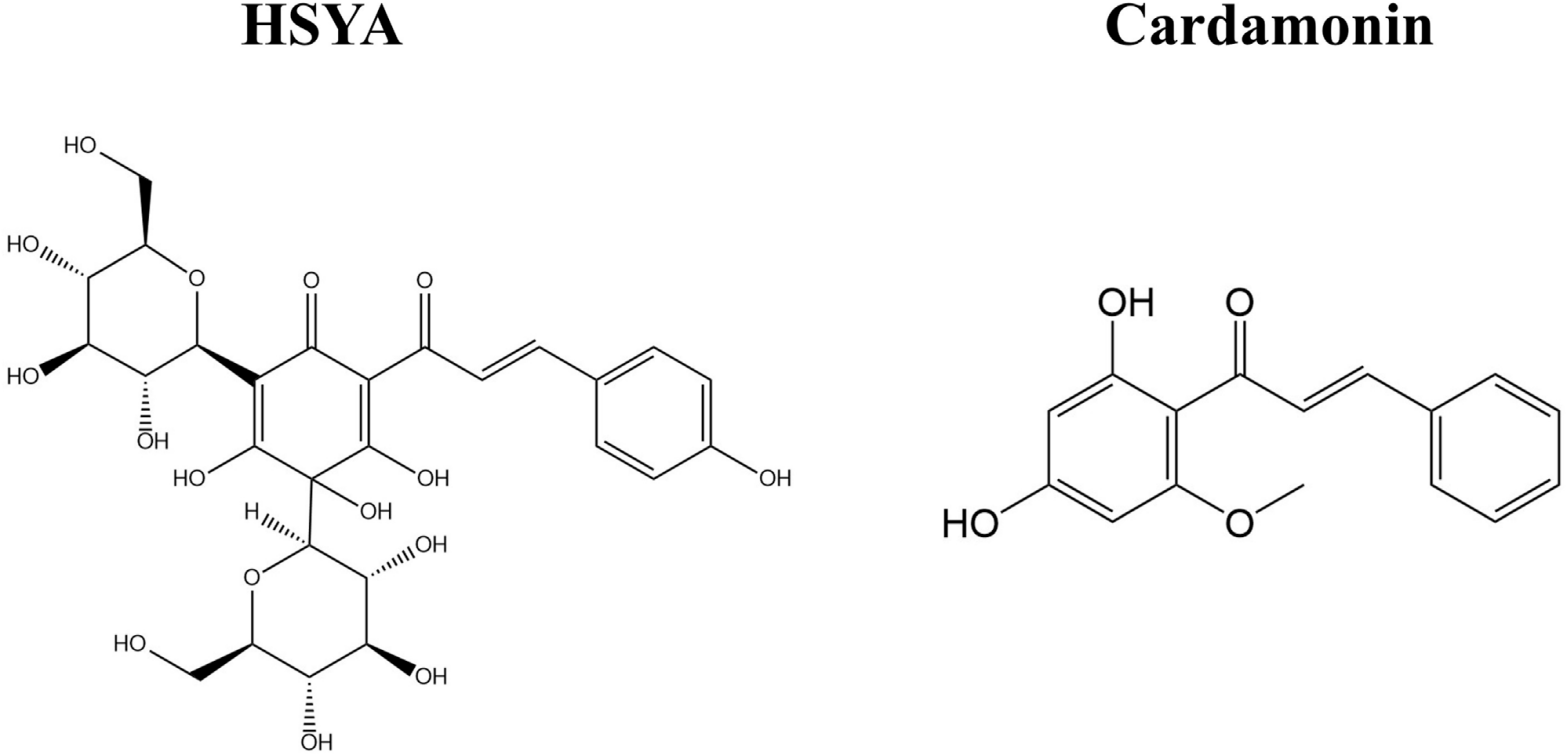
The structure of HSYA and cardamonin

The most abundant constituents of safflower are flavonoids and fatty oil, followed by phenylethanoid glycosides, coumarins, steroids, and polysaccharides (Xian et al., 2022). Among these, flavonoids are the characteristic bioactive constituents (Zhang et al., 2016). SY refers to a class of major bioactive compounds in safflower with a flavonoid structure, including safflower yellow A (SYA), safflower yellow B (SYB), and HSYA. Notably, HSYA constitutes 85% of SY and is considered the main bioactive component pigment of safflower (Wu et al., 2021). Natural pigments are derived from a wide variety of sources in nature. Structurally, they can be broadly classified into isoprene derivatives, polyphenols, ketone derivatives, tetrapyrrole derivatives, and quinone derivatives (Eleonora et al., 2023). HSYA is a unique flavonoid compound in safflower, belonging to the class of quinochalcone compounds (Xian et al., 2022). These compounds, characterized by a distinctive C-glycosylated cyclohexanonedienol molecular structure, are found exclusively in safflower. They exhibit significant efficacy in managing cardiovascular diseases (Zhao et al., 2020). Research has also indicated that chalcone compounds possess anticancer properties (Mendez-Callejas et al., 2023; Ouyang et al., 2021). For example, cardamonin derived from *Alpinia katsumadai Hayata* with a chalcone structure has anticancer activity against breast and ovarian cancer (Jin et al., 2019), suggesting that quinochalcone compounds may also have anticancer potential (Figure 2). There have been more and more reports of natural pigments with anticancer effects, such as carotenoids (isoprenoid polymers) (Antoine et al., 2022), anthocyanins (polyphenols) (Wallace and Giusti, 2019), and green tea polyphenols EGCG (polyphenols) (Singh et al., 2011). Compared to these natural pigments, HSYA offers distinct advantages in terms of targeting and its multiple anticancer mechanisms. Specifically, HSYA exhibits stronger anti-tumor effects, including inhibition of proliferation, migration, and angiogenesis, compared to anthocyanins and carotenoids (Rodriguez-Amaya, 2019). Additionally, HSYA regulates the tumor microenvironment, reduces tumor angiogenesis, and addresses drug resistance, suggesting its potential across various cancer types (e.g., lung, breast, and liver cancers) (Yang et al., 2015). In contrast, EGCG predominantly targets gastrointestinal cancers (Peng et al., 2024). Regarding targeting, HSYA effectively targets cancer cells and limits tumor spread and metastasis by modulating key signaling pathways (e.g., p53, NF-κB, VEGF, *etc.*) (Li et al., 2014), Although EGCG has shown notable anticancer effects in clinical treatments for gastrointestinal cancers, challenges such as drug resistance remain (Mereles and Hunstein, 2011; Singh et al., 2011). On the other hand, HSYA requires further clinical studies and optimization of its pharmacokinetics (Chen Q. et al., 2022) (Table 1).

**TABLE 1:** The anti-cancer characteristics of natural pigments.

Name	Categories	Anti-proliferation Anti-metastasis and invasion Anti-angiogenesis	Targeting ability	Preclinical and clinical research	Future research potential	References
HSYA	Ketone derivatives	Promotes tumor cell apoptosis by regulating the cell cycle and up-regulating p53, p21 Inhibits MMPs Inhibits VEGF, limiting tumor angiogenesis	Regulate tumor micro-environment	Anti-cancer effects in multiple *in vitro* and animal models, limited clinical research	Further optimization of pharmacokinetics	(Yang et al., 2015) (Li et al., 2014)
Carotenoids	Isoprenoid polymer	Moderate inhibitory effect, improves immune response Weak Not significant	Mainly through immune system enhancement, indirectly affects tumors	Widely studied, showing some effects in certain cancer types	Suitable as a dietary supplement, weak anti-cancer effect	(Antoine et al., 2022)
Anthocyanins	Polyphenols	Moderate inhibitory effect Moderate, through antioxidant and anti-inflammatory effects Some effect, but weaker than HSYA	Primarily acts on antioxidant and anti-proliferation, lower targeting ability	Mostly *in vitro* studies, clinical effects need further validation	Further exploration of effects and mechanisms in various cancers needed	(Wallace and Giusti, 2019)
EGCG	Polyphenols	Inhibits tumor cell proliferation through multiple mechanisms, particularly in gastric and lung cancer	Precisely target cancer cells through specific pathways (e.g., MAPK, PI3K/Akt)	Several clinical studies show significant anti-cancer effects, especially in gastrointestinal cancers	Potential as a monotherapy or combination therapy issues with resistance	(Peng et al., 2024) (Mereles and Hunstein, 2011) (Singh et al., 2011)

HSYA (C_27_H_32_O_16_) is an orange-yellow powdery substance highly soluble in water. It is poorly lipophilic and virtually insoluble in organic solvents such as chloroform, benzene, and ethyl acetate (Xue et al., 2021). Furthermore, HSYA is structurally unstable and rapidly degrades under strong acidic and alkaline conditions. Consequently, it is unstable when administered orally as a single compound in the presence of gastric acid. Due to the presence of phenolic hydroxyl groups, HSYA exists in a protonated form in natural or alkaline aqueous solutions, significantly impairing its transmembrane transport and resulting in low bioavailability. The absolute oral bioavailability (F) of HSYA administered by gavage is only 1.2% (Zhang et al., 2006). It does not tolerate high temperatures and degrades readily at water temperatures greater than 60°C. For centrally-targeted drugs, the ideal pKa range is 5–10, as basic and amphoteric drugs are more likely to cross the blood-brain barrier (BBB). HSYA is predicted to have a pKa value of 4.50 ± 1.00, but experimental evidence indicates that HSYA is more likely to cross a damaged BBB, making it difficult to pass through a normal BBB (Sheng et al., 2020). Additionally, HSYA degrades under light exposure and must be stored away from light. The application of small amounts of ethylenediamine tetra-acetic acid and ascorbic acid has been shown to improve its stability (Tang et al., 2021).

Various methods of extraction have been employed for the extraction of HSYA, including water immersion, alcohol extraction, ultrasonic extraction, and microwave extraction (Table 2). Water immersion is the most commonly used traditional method, as it is simple to operate, easy to control, and cost-effective. However, its yield is relatively low, typically around 0.066%, and it requires a large amount of raw material, which can easily degrade the HSYA due to high temperature, alkaline conditions, and light exposure (Zhao et al., 2020). With the continuous refinement of extraction techniques, the ultrasonic extraction method, conducted at low temperatures, has shown stable and reproducible results. Despite optimization of solvent volume and extraction time, the yield remains around 1.7% (Jin, 2010). More recently, Xue employed a rapid and simple microwave extraction method to improve HSYA production, achieving a yield of 6.96% by using a solid-to-liquid ratio of 1:100, maintaining a temperature of 70°C, and conducting three cycles within 20 min (Hong et al., 2015). However, this method requires a large volume of solvent and can result in variable composition. Notably, Li et al. reported the highest extraction efficiency of HSYA (14.564%) using DMSO as the solvent. However, due to the excellent solubility of DMSO, the HSYA extract contained high levels of impurities, which reduced its purity (Li et al., 2016). Therefore, this method is not widely used (Yao and Matosevic, 2021).

**TABLE 2:** The development of extraction methods examples of HSYA.

Method	Conditions	Yield	Advantage	Disadvantage	References
Water immersion method	Raw materials shade dried and powdered 12.5 times the amount of distilled water (60°C, 30 min)for 3 cycles; evaporation under reduced pressure; dissolved in 10% ethanol (1,000 mL) evaporated to dryness under vacuum	0.07%	simple operation, easy to control the extraction process, economic	low yield and high consumption of raw materials, ingredients easily destroyed	(Zhao et al., 2020)
Ethanol extraction method	6 times the amount of 75% aqueous ethanol (12 h) for 10 cycles; concentration to dryness *in vacuo* at 55°C; resolved with water and extracted by petroleum ether and ethyl acetate five times	0.58%	Simple operation, stable effect, high HSYA transfer rate, leachate yield and purity	low yield	(Li et al., 2016)
Flash extraction method	40 times the amount of distilled water (2.5 min, 90 V) and filtered	1.36%	the active ingredients destroyed by heat, short extraction time, high efficiency	medium yield	(Jin, 2010)
Ultrasonic extraction method	18 times the amount of 30% aqueous ethanol, ultrasonic extraction (1 h)	1.73%	process stable and reproducible	medium yield	(Jin, 2010)
MAS-I microwave extraction method	100 times the amount of distilled water (70°C, 20min) for 3 cycles	6.96%	rapid and easy to implement; higher yield	high solvent volume requirement; variable composition	(Hong et al., 2015)
DMSO extraction method	Stirred 14 times the amount of DMSO at R.T. to avoid light for 30 min, impurity removal, filtered Add DMSO to soaking, heating extraction (seal, 80°C, 50 min), and filter Then, 12 times the amount of DMSO was added to the residue, heating extraction (seal, 80°C, 50 min) Filtered and combined the filtrate Three times the amount of butyl acetate was added to the filtrate and centrifuged Washed hot, cold, and ultrafiltration the precipitate with ethanol, dried	14.56%	Simple, economic, environmentally friendly, higher yield	high impurity content, low purity of extracts	(Li et al., 2016)

## 3 Pharmacological action and molecular mechanism of HSYA

**FIGURE 3:**
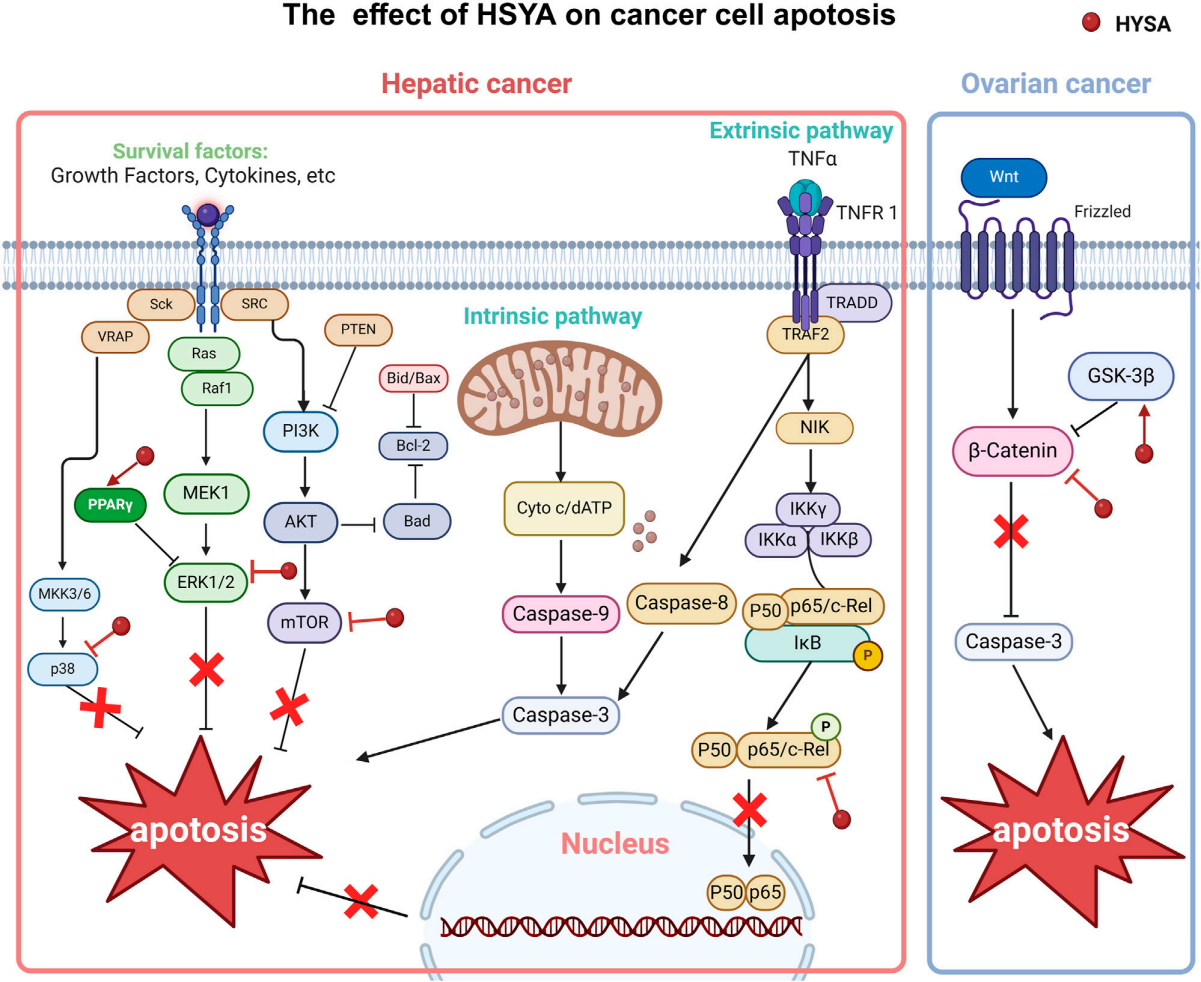
The effect of HSYA on cancer cell apotosis in hepatic cancer and ovarian cancer.

**FIGURE 4:**
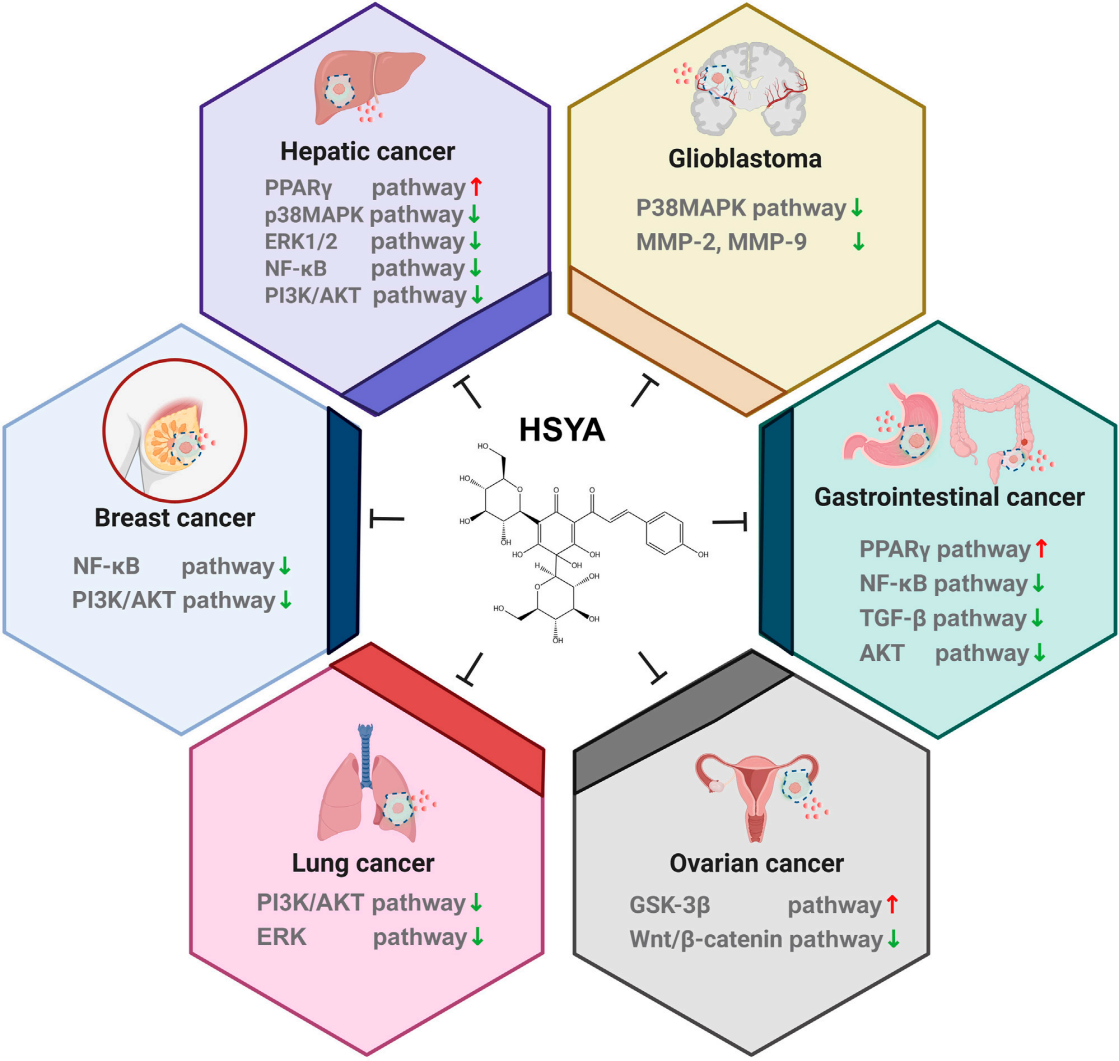
The major proven regulatory pathways of HSYA on various cancer.

The SY injection, with HSYA as the main ingredient, is one of the few Traditional Chinese Medicine (TCM) injections used as a clinical therapeutic agent, and its development prospects are considerable (Chen Y. et al., 2022). Previous studies have focused primarily on its effects on cardiovascular and cerebrovascular diseases (Sun et al., 2023). In the last 10 years, many studies analyzed the effect of HSYA on various cancer cells and revealed that HSYA has a good anticancer effect; thereby, indicating that it is a promising therapeutic agent with anticancer effects (Ao et al., 2018; Chen Q. et al., 2022). Till date, HSYA has made much progress in the study of hepatocellular carcinoma compared to other cancers. This section provides detailed pharmacological actions and mechanisms of the HSYA signaling pathways (Table 3; Figures 3, 4).

**TABLE 3:** Pathways and targets of HSYA in various cancer types.

Disease	Model	Administration	Outcome	Mechanism/Pathways	References
Hepatic cancer	H22 tumor-bearing mice	1.125 mg/kg 2.25 mg/kg	i.p	↓ VEGF A, bFGF,VEGF 1; suppress ERK1/2 phosphorylation and inactivate NF-κB ↓ cyclinD1, c-myc, c-Fos	ERK1/2 NF-κB	(Yang et al., 2015)
	Hep-G2 cells H22 tumor-bearing mice	80 μmol/L 1.125 mg/kg 2.25 mg/kg	i.p	Suppression of p38 activation ↓ MMP-2, MMP-9 and COX-2	p38MAPK/ATF-2	(Zhang et al., 2019)
	Hep-G2 cells	2 μmol/L	NA	↑ Beclin 1 and inhibiting the phosphorylation of ERK; autophagy activation	Ras/Raf/ERK	(Chen et al., 2020a)
	Huh7 cells Hep-G2 cells Huh7 tumor-bearing mice	160 μmol/L 1.125 mg/kg 2.25 mg/kg	i.p	Impairing the lysosomal acidification and LAMP1 ↓ Inhibiting AKT and mTOR; autophagosome accumulation, blocking the late-phase of autophagy flux;	PI3K/AKT/mTOR	(Chen et al., 2020b)
	SMMC-7721 cells H22 Induced Lung Metastasis Mice	1.5 μmol/L 0.57 mg/kg 1.13 mg/kg 2.25 mg/kg	i.p	Activation of PPAR-γ, inhibition of MMP-2; degradation of ECM and EMT inhibition	PPAR-γ	(Ma et al., 2017)
	Hepa1-6 *in situ* mice	0.57 mg/kg 1.13 mg/kg 2.25 mg/kg	i.p	↓ FOXP3-expressing Tregs and *ROR*γt in tumor tissue ↓ CD4^+^CD25^+^FOXP3^+^ Tregs/CD4^+^T lymphocytes in the spleen without weight loss compared to cisplatin	Tumor immune micro-environment	(Ma et al., 2019)
Lung cancer	A549 cells H1299cells	5 μmol/L 10 μmol/L 20 μmol/L	NA	↓ ERK、PI3K、AKT、mTOR ↓ TNF-α, IL-6, IL-1β, and IL-10 ↓ EMT	PI3K/AKT/mTOR ERK/MAPK	[Jiang et al., 2015]
Breast cancer	MCF-7 cells	50 μg/mL 100 μg/mL	NA	Blocking the nuclear translocation of NF-κB/p65; ROS generation, loss in mitochondrial membrane potential ↑ Bax and p53, ↓ Bcl-2 and cyclin D1 cyto-c release from mitochondrial to cytosol caspase-3 activation	NF-κB, mitochondrial apoptotic	(Li et al., 2014)
	MBA - MD - 231 cells (SY)	0.19 mg/mL 0.39 mg/mL 0.75 mg/mL	NA	↓ MMP-9 and p-Src (×Src dependent cytoskeleton rearrangement) prevent invadopodia formation		(Fu et al., 2016)
	MCF-7 cells (HSYB)	10 μg/mL 20 μg/mL 30 μg/mL	NA	↓ PI3K-AKT ↓ cyclin D1, cyclin E, and CDK2 (arrested cell cycle at the S phase)	PI3K-AKT	[Qu et al., 2019]
Ovarian cancer	HO-8910PM cells; HO-8910PM tumor-bearing mice	200μmol /L; 50mg /kg	p.o	Promoting the expression of menin; resulting in β-catenin degradation;↓ MMP-7 and Survivin	Wnt/β-catenin	[Gong and Zhou, 2019]
	SKOV3 cells; SKOV3 tumor-bearing mice	50, 100, 200μmol/L; 12.5, 25.0, 50.0 mg/kg	p.o	GSK-3β activation; resulting in β-catenin degradation in the nucleus; the expression of many oncogenes downstream	GSK-3β	[Gong et al., 2020]
	SKOV-3 cells and CD4^+^T cell co-culture	2μg/ml	NA	Inhibiting the activation of TLR8; ↓ the percentage of Th1 cells and Th17 cells; ↑ the polarization of Th2 cells, thus regulating the polarization of CD4^+^T cells	tumor immune micro-environment.	[Hu et al., 2021]
Gastric cancer	BGC-823 cells nude mice model	0.028 g/L	i.p	The micro-vessel density (MVD) decrease		[Liu et al., 2018]
	BGC-823 cells	100μmol/L	NA	PPARγ activation; block G0/ G1 phase to S phase, ↑caspase-3	PPARγ	[Liu et al., 2018]
Esophageal Cancer	EC cells	20μmol/L	NA	NF-κB inhibition; ↓ ICAM_1_, MMP9, TNF-α, and VCAM_1_	NF-κB	[Chen et al., 2020b]
Colorectal Cancer	HT-29 cells	0.2μg/mL 2μg/mL 20μg/mL	NA	↓ TGF-β, Smad2 and α-SMA protein , inhibition of TGF-β Signal pathway activation: G0/G1 phase cells increase ↑ E-cadherin protein, ↓ Vi and FN protein inhibit HT-29 EMT	TGF-β	[Wen et al., 2017]
	HCT116 cell	25μmol/L 50μmol/L 100μmol/L	NA	↑ PPARγ to inhibit Akt activation Regulating PCNA, Bax, Bcl-2, cleaved-caspase3, E-cadherin, N-cadherin, vimentin	PPARγ/ PTEN/Akt	[Su and Lu 2021]
Glioblastoma	U251cells	25 mmol/L 100 mmol/L 400 mmol/L	NA	↓ MMP2 MMP9		[Yuan and Lou, 2016]
	U251cells	25μmol/L 100μmol/L 400μmol/L	NA	suppression of p38 activation; ↓ MMP-2, MMP-9	P38 MAPK	[Wan, 2018]
	LN-229 cells U-251MG cell	25μmol/L 50μmol/L 100μmol/L	NA	↑ γH2AX to cause DNA damage; silencing MYC expression to inhibit MRN complex, leading to DNA repair deficiency	DNA damage	[Tang et al., 2021]

### 3.1 Hepatic cancer

HSYA can inhibit the secretion of angiogenesis factors (VEGF A and bFGF) and VEGF 1, suppressing ERK1/2 phosphorylation, and inactivate NF-κB by inhibiting nuclear translocation. These effects are mediated by regulating P65 expression levels both inside and outside the nucleus (upregulation within the nucleus), inhibiting IκB phosphorylation, and preventing cytoplasmic degradation of IκBα. This results in a decreased expression of proliferation-related genes, including cyclin D1, c-Myc, and c-Fos. Studies have shown that HSYA inhibits angiogenesis, hepatoma cell viability, and tumor development by modulating the ERK/MAPK and NF-κB pathways in H22 tumor-bearing mice (Yang et al., 2015).

HSYA effectively lowered the production of MMP-2, MMP-9, and COX-2 by inhibiting the p38MAPK/ATF-2 signaling pathway. This finding explains the observed inhibition of the viability, proliferation, and migration of HepG2 cells during HSYA treatment, which can be attributed to the suppression of p38 activation (Zhang et al., 2019). Butein, a natural chalcone similar in structure to HSYA, promotes apoptosis *in vitro* by inhibiting NF-κB, p38 MAPK, and JNK pathways (Agnieszka et al., 2013).

**FIGURE 5:**
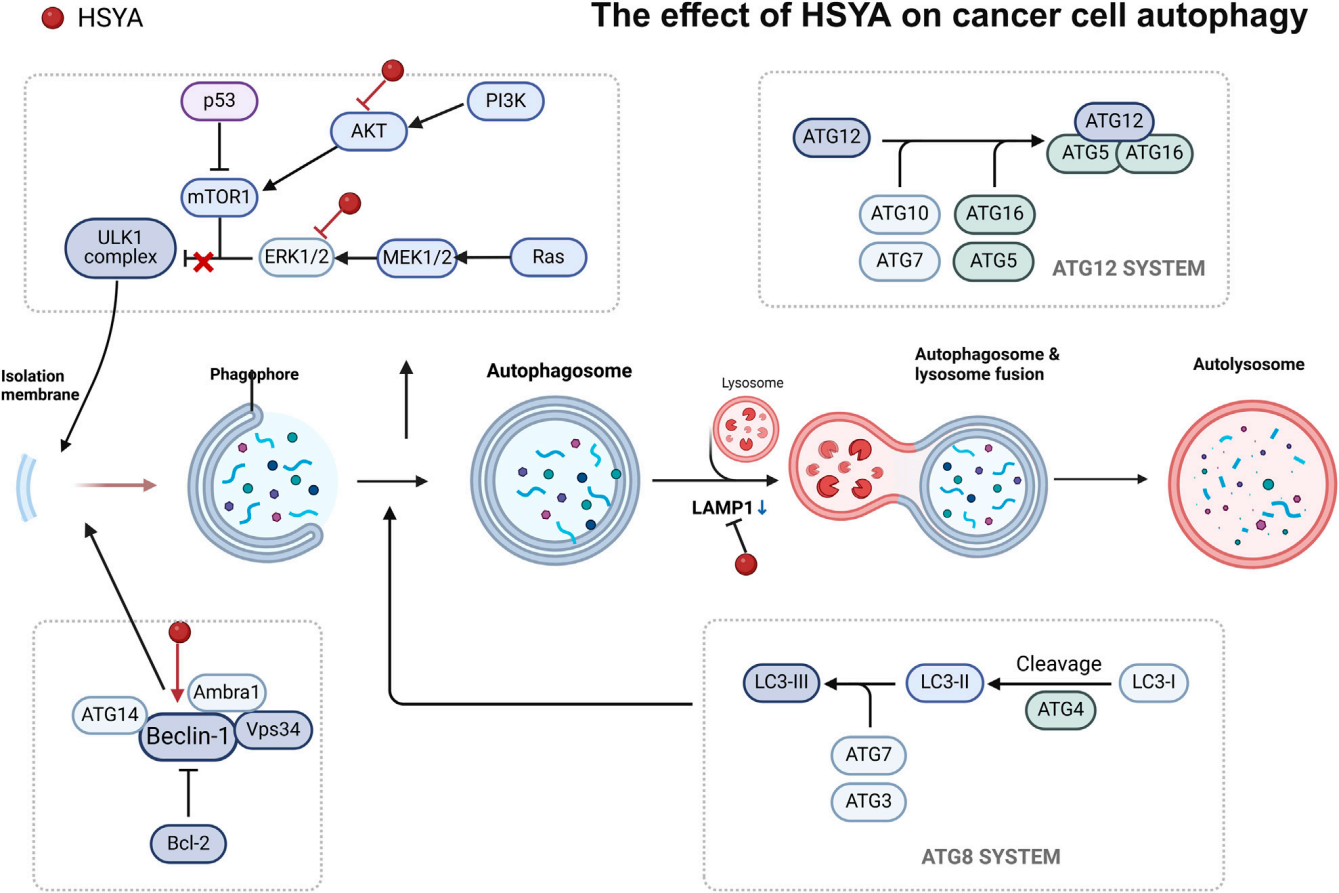
The effect of HSYA on cancer cell autophagy in hepatic cancer.

Several studies have reported a correlation between HSYA expression and autophagy. In this study, HSYA activated autophagy in HepG2 cells by upregulating beclin one expression and suppressing ERK phosphorylation (Chen X. et al., 2020; Zhang et al., 2016). The Ras/Raf/ERK signaling pathway can induce autophagy; therefore, ERK may be a target of the HSYA anti-liver cancer effect of HSYA. Therefore, ERK can be used as a target for treating pancreatic cancer. However, a single method of autophagy activation has limited therapeutic effects on tumors (Amaravadi et al., 2016). Clinical studies have shown that the activation of autophagy can inhibit tumor growth more effectively by blocking autophagic flux (Fan et al., 2022) (Figure 5).

Moreover, HSYA induced an autophagic response and autophagosome accumulation by blocking autophagic flux. This disrupts the metabolic cycle of tumor cells, reduces their adaptability, and leads to apoptosis. Recent studies have shown that HSYA impairs late-stage autophagic flux by inhibiting lysosomal acidification and downregulating LAMP1 expression, leading to apoptosis via the PI3K/AKT/mTOR signaling pathway (Chen Z. et al., 2020). PI3K/Akt/mTOR signaling inhibits autophagy. However, the activation of PTEN and AMPK by P53 promoted autophagy by blocking PI3K and mTOR activation, suggesting that HSYA may enhance P53 activation (Chen et al., 2019) (Figure 5).

**FIGURE 6:**
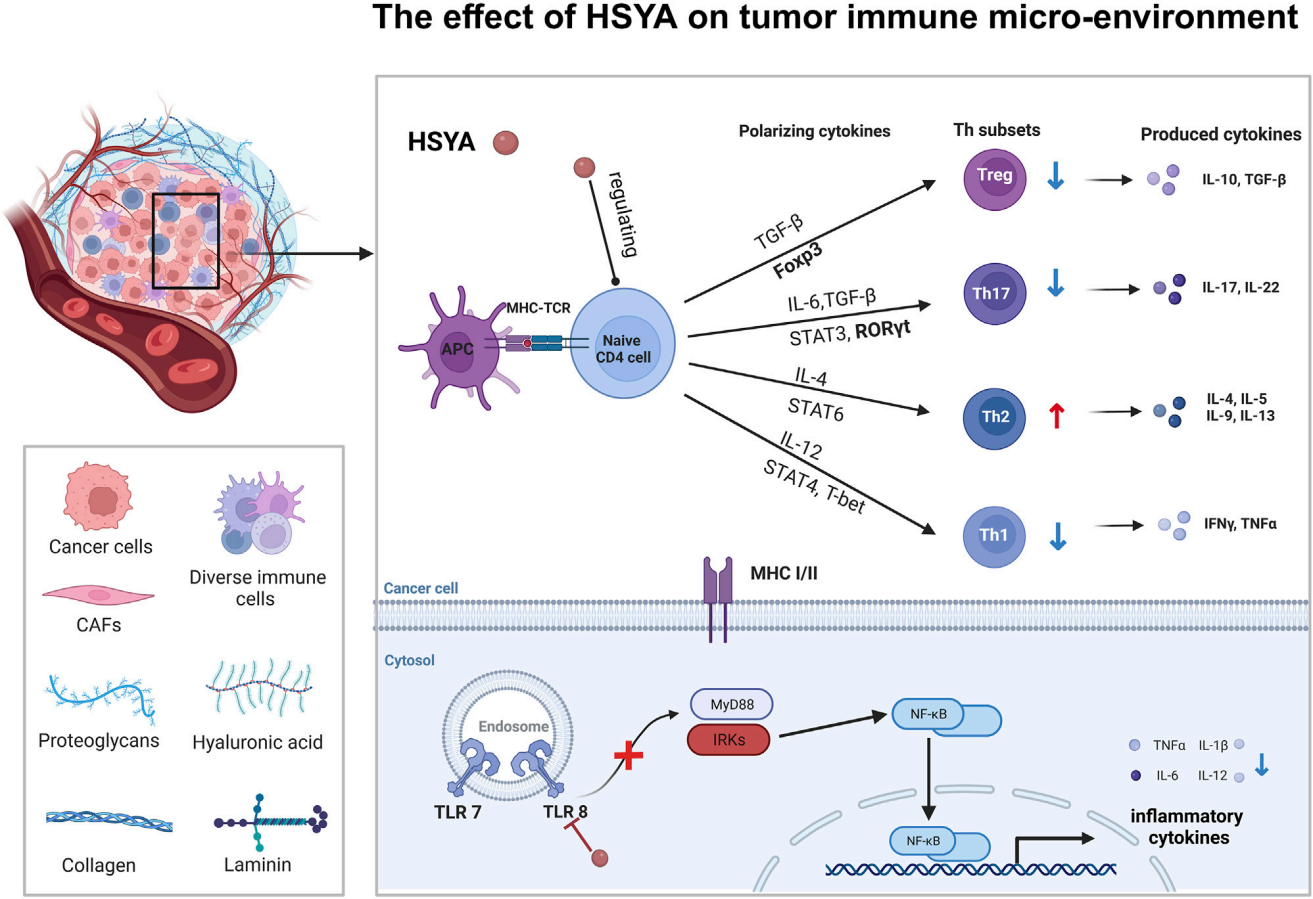
The effect of HSYA on tumor immune micro-environment in hepatic cancer and ovarian cancer.

HSYA has been reported to activate PPARγ, inhibiting oxidative stress-induced liver fibrosis (Wang et al., 2013). WU *et al.* demonstrated that HSYA could induce extracellular matrix (ECM) degradation and inhibit epithelial-mesenchymal transition (EMT) by activating PPARγ and inhibiting MMP-2 in SMMC-7721 cells (Ma et al., 2017). All these may indicate that HSYA may also be a PPARγ agonist and then antagonized TGF-β, resulting in down-regulating E-cadherin expression and MMPs and EMT inhibition in the final. Studies have indicated that the activation of PPARγ occurs subsequent to the binding of a ligand. Studies have also shown that PPARγ is activated after ligand binding and upregulates PTEN expression in colorectal cancer (CRC) cells. PTEN mediates cellular biological processes by inhibiting the PI3K/Akt signaling cascade (Lu et al., 2012). HSYA can also regulate the tumor immune microenvironment to interfere with tumor progression. In the process of the Treg differentiation, TGF-β and IL-2 trigger iTregs to FOXP3. iTregs are phenotypically CD4^+^CD25^+^CD127 low/−, and generally FOXP3^+^. *In vivo* study, HSYA can participate in this process and downregulate the level of FOXP3-expressing Tregs and Rort γ in the liver tumor tissue of the Hepa1-6 mice model. HSYA inhibits the levels of the CD4^+^CD25^+^FOXP3^+^ Tregs/CD4^+^T lymphocytes in liver cancer cells. Surprisingly, HSYA substantially inhibited tumor growth without loss of body weight in Hepa1-6 mice compared to cisplatin (Ma et al., 2019), which could be developed into a new drug treatment strategy for liver cancer with few side effects (Figure 6).

### 3.2 Lung cancer

Research on HSYA’s effects of HSYA on lung cancer is limited (Xia et al., 2022). JIN *et al.* found that HSYA attenuates pulmonary fibrosis *in vivo* model in 2016 (Jin et al., 2016). In pulmonary fibrosis, HSYA effectively inhibit cell proliferation induced by TGF-β1. Currently, the research conducted by HSYA on the pulmonary system has mostly focused on fibrosis. The partial protection against renal cell fibrosis by HSYA is attributed to the inhibition of the TGF-β1/smad3-mediated EMT signal pathway (Jin et al., 2016). HSYA is also effective for the treatment of lung cancer.

HSYA inhibits LPS-induced expression of inflammatory cytokines by blocking the PI3K/Akt/mTOR and ERK/MAPK signaling pathways. This inhibition resulted in reduced migration, invasion, and EMT, ultimately promoting the apoptosis of A549 and H1299 cells (Jiang et al., 2019).

### 3.3 Breast cancer

Safflower, a source of HSYA, has been extensively studied in breast cancer. SiHong Tang, a traditional Chinese medicinal formulation containing safflower, was been investigated for its effects on breast cancer (Jiang et al., 2021). SY, the primary active component of safflower extract, includes SY A, SY B, and HSYA. SY substantially inhibits the migration of MBA-MD-231 cells and lung metastasis of breast cancer cells *in vitro* in a 4T1 luciferase cell-based *in vivo* model. SY treatment also reduced EGF-stimulated invadopodia formation and MMP-9 and Tyr416 expression. Furthermore, the number of circulating tumor cells in the pulmonary capillaries was decreased. Thus, SY’s anti-metastasis effect of safflower yellow on breast cancer primarily prevents invadopodia formation via Src-dependent cytoskeletal rearrangement (Fu et al., 2016). To clarify, the involvement of HSYA in breast cancer, Liu *et al.* conducted a study that demonstrated that HSYA triggered the upregulation of Bax and p53 in breast cancer cells. Additionally, HSYA was found to induce the generation of reactive oxygen species (ROS) and promote the release of cytochrome c (cyto-c) from the mitochondria into the cytosol, causing a decline in the mitochondrial membrane potential and activating caspase-3. These effects were attributed to inhibiting the nuclear translocation of the NF-κB/p65 pathway in MCF-7 cell vitro study. Consequently, it is plausible that HSYA triggers apoptosis in breast cancer cells via the mitochondrial apoptotic pathway (Li et al., 2014).

Hydroxysafflor yellow B (HSYB), an isomer of HSYA, has also been investigated. The combined treatment with HSYB and DOX (an established chemotherapeutic drug for breast cancer) increased ROS generation and promoted cytochrome c release in MCF-7 cells, reduced cell proliferation and induced apoptosis. HSYB also downregulates cyclin D1, cyclin E, and CDK2 to arrest the MCF-7 cell cycle in the S phase, leading to apoptosis via the PI3K-AKT pathway (Qu et al., 2019). Cardamonin, which has a similar structure to HSYA, has been shown to cause EMT reversal of BT-549 cells to inhibit the invasion of breast cancer cells through Wnt/β-catenin signaling (Shrivastava et al., 2017), and the above shows that HSYA has great potential for breast cancer research.

### 3.4 Ovarian cancer

Both *in vivo* and *in vitro* studies have shown that HSYA inhibits the growth of ovarian cancer cell growth and promotes apoptosis by upregulating menin expression. This leads to β-catenin degradation, thereby reducing the expression of the downstream oncogenes, MMP-7 and Survivin (Gong and Zhou, 2019). Several studies have further elucidated that HSYA inhibits the phosphorylation of GSK-3β at the Ser9 site, which subsequently promotes the phosphorylation of GSK-3β at the Tyr216 site. This results in the activation of GSK-3β and subsequent destruction of β-catenin. As a result, the nuclear localization of β-catenin is reduced, leading to a concomitant decrease in the expression of several downstream oncogenes. This process inhibited the proliferation of ovarian cancer cells and induced cell death (Gong et al., 2020). SY inhibits the proliferation, invasion, and migration of SKOV-3 ovarian cancer cells by blocking the TGF-β1 pathway. Its mechanism may involve the inhibition of intracellular ROS levels and the attenuation of EMT mediated by SY (Wang and An, 2021). In contrast, cardamonin exerts its anticancer effect in ovarian cancer cells (SKOV3 and PDC cells) model by arresting the G2/M phase and cell apoptosis through inhibition of NF-κB and mTOR pathways (Ruibin et al., 2020).

HSYA also inhibits ovarian cancer growth by modulating the tumor’s immune microenvironment of tumors. HSYA reduces the proportion of Th1 and Th17 cells in SKOV-3 cells by blocking the TLR8 signaling pathway. This inhibition leads to a decrease in the polarization of Th1 and Th17 cells, while simultaneously promoting the polarization of Th2 cells and influencing the polarization of CD4^+^ T cells. These findings suggested that the regulation of CD4^+^ T cell polarization and immune cytokine production in ovarian cancer by HSYA may occur through the TLR8 signaling pathway, leading to alterations in tumor metabolism and enhancement of immune function within the tumor micro-environment (Hu et al., 2021) (Figure 6).

### 3.5 Gastrointestinal cancer

HSYA reduces micro-vessel density (MVD) in tumor tissues in a BGC-823 cell xenograft model in nude mice. MVD is strongly correlated with tumor growth, invasion, metastasis, and prognosis, indicating that HSYA may impede the invasion and metastasis of human gastric adenocarcinoma. A specific dose of HSYA effectively suppressed excessive proliferation of vascular endothelial cells, decreased the proliferation of BGC-823 cells, and induced apoptosis by arresting the cell cycle. HSYA exerts an anti-tumor effect by inhibiting both vascular endothelial cells and tumor cell growth, while promoting tumor cell death. In an *in vitro* study, HSYA treatment resulted in a shift in BGC-823 cells from the G0/G1 phase to the S phase. However, this transition was inhibited by activating PPAR, leading to cell cycle arrest and apoptosis (Liu et al., 2018).

HSYA have inhibitory effects on the expression of ICAM_1,_ MMP9, TNF-α, and VCAM_1,_. Additionally, it promotes the expression of phospho-NF-κB p65 and phospho-IκBα. However, no subsantial changes in the expression of P65 and IκBα have been reported. HSYA inhibits the proliferation, invasion, and migration of EC cells, which is partially mediated by the regulation of the NF-κB signaling pathway. This regulation leads to the induction of apoptosis in EC (Chen X. et al., 2020).

*In vitro* studies have shown that HSYA upregulates the expression of E-cadherin and downregulates the expression of vimentin (Vi) and fibronectin (FN). HSYA inhibits HT-29 EMT, likely by suppressing TGF-β signaling pathway activation through the decreased expression of TGF-β, Smad2, and α-SMA proteins. Consequently, the apoptotic rate of HT-29 cells increased through G0/G1 phase arrest. Overall, HSYA inhibits the proliferation, invasion, and metastasis of HT-29 cells (Wen et al., 2017). HSYA substantially reduced colorectal cancer HCT116 cell viability but had no effect on normal cell HIEC. HSYA also inhibited cell proliferation, migration, and invasion but promoted apoptosis of HCT116 cells by up-regulating PPARγ to inhibit Akt activation. In summary, HSYA has the potential to demonstrate anticancer properties in CRC by activating the PPARγ/PTEN/Akt signaling pathway. Furthermore, the research revealed that the administration of PPARγ antagonist and the process of PPARγ knockdown did not entirely impede the anticancer properties of HSYA, which suggested that other pathways were involved in HSYA-mediated CRC inhibition (Su and Lv, 2021).

### 3.6 Glioblastoma

Several studies have reported that HSYA exhibits neuroprotective properties in rat SCI models of spinal cord injury. These studies have shown that HSYA effectively controls oxidative stress, prevents the production of pro-inflammatory molecules, and lowers neuron apoptosis through the NF-κB pathway. These findings suggest that HSYA holds promise as a neuroprotective agent (Pei et al., 2017). HSYA (30 mg/kg) plays the role of anti-traumatic brain injury (TBI rat model) by improving the integrity of BBB, and its mechanism may be related to the downregulation of TLR4/NF-κB pathway to play the role of antioxidation, anti-inflammatory, anti-apoptosis, etc (Ren et al., 2022). Furthermore, we found that HSYA inhibited the proliferation, migration, and invasion of glioma U251 cells (Yuan and Lou, 2016), and its mechanism may be related to the downregulation of the expression of MMP-2 and MMP-9. MMP-2 and MMP-9 are gelatinases in MMPs (zinc ion-dependent proteases which can degrade extracellular matrix proteins) that can degrade collagen IV, laminin, and fibronectin, the main components of the perivascular basement membrane, and facilitate tumor cells to invade surrounding tissues along the basement membrane. High invasiveness of gliomas is associated with the expression activity of MMP-2 and MMP-9 (Wan, 2018).

HSYA induced the cell apoptosis of glioma cells LN-229 and U-251MG cells by enhancing the expression of γH2AX to cause DNA damage. However, the DNA repair reaction was normal in HSYA-treated glioma cells. Interestingly, silencing MYC expression can enhance the sensitivity of HSYA to gliomas by inhibiting the MRN (MRE11RAD50-NBS1) complex, resulting in DNA repair defects. Inhibition of MYC results in the suppression of the DNA damage response through the control of NBS1 (DNA repairing protein), ultimately causing defects in DNA repair (Tang et al., 2021). Studies have shown that PTEN plays a crucial role in DNA repair, particularly in homologous recombination repair (HR)(Han et al., 2024). PTEN can directly interact with the MRE11 protein in the MRN complex, thereby affecting MRE11 activity and thereby influencing DNA repair efficiency. Through its phosphatase activity, PTEN inhibits the PI3K/Akt signaling pathway, which regulates the DNA damage response Activation of the PI3K/Akt pathway suppresses HR repair, while PTEN negatively regulates this pathway to promote HR repair (Ming and He, 2012). PTEN modulates MRN complex function during this process (Michael et al., 2021). Furthermore, as mentioned earlier, this suggests that HSYA can affect the PTEN/AKT/mTOR pathway to inhibit MRN, thereby affecting HR repair.

From the above results, we can see that HSYA has been studied extensively in liver cancer. Notably, HSYA mainly induces apoptosis in hepatocellular carcinoma cells by regulating PI3K/AKT/mTOR pathways, and interestingly Wnt/β-catenin pathway, which has not yet to be elucidated in hepatocellular carcinoma, has been involved in the study of ovarian carcinoma (Figure 3), and in autophagy, HSYA regulates autophagy of hepatocellular carcinoma cells through regulating In terms of cellular autophagy, HSYA regulates autophagy in hepatocellular carcinoma cells through the regulation of ERK, AKT pathway and Beclin-1 (Figure 5); in terms of regulating the tumor immune microenvironment, HSYA regulates the Naïve cell differentiation in hepatocellular carcinoma cells, and in ovarian carcinoma cells, HSYA can also inhibit TL8 and thus regulate inflammatory factors (Figure 6).

## 4 Pharmacokinetic progression

Absorption: HSYA is classified as a Biopharmaceutical Classification System (BCS) class III drug. To date, there are two primary routes of administration: oral and intravenous. Results from the rat intestinal loop assay indicated that HSYA is not absorbed in the stomach but is rapidly absorbed in the intestinal lumen (Zhang et al., 2006). After saffron extract was administered via gavage to rats, HSYA was primarily absorbed in the small intestine (Yi et al., 2007). Pharmacokinetic studies show that the oral absolute bioavailability (F) of HSYA is only 1.2%, and its blood concentration-time curve exhibits a bimodal pattern in rats following oral gavage.

Distribution: The plasma protein binding rate of HSYA was found to be approximately 50% after 48 and 72 h of equilibrium dialysis, suggesting that its plasma protein binding is low. This indicates that HSYA’s efficacy is not easily enhanced by the competitive displacement of plasma protein binding sites during co-administration of other drugs (Chu et al., 2006). Following tail vein injection of safflower lyophilized powder, HSYA was distributed to various organs, including the blood, kidney, liver, lung, heart, and spleen, with the highest area under the curve (AUC) observed in the liver, kidney, spleen, heart, and lungs. Notably, HSYA was not detected in the brain (Li T. et al., 2021).

Metabolism: HSYA undergoes reduction and hydrolysis by hepatic microsomal drug-metabolizing enzymes, generating phase I metabolites through hydroxylation, methylation, dehydration, hydrogenation, and hydration. Phase II metabolism includes acetylation, glucuronidation, and additional reactions such as methylation and glucuronide conjugation (Liang et al., 2018). In rats, following oral gavage, eight metabolites were detected in plasma, bile, urine, and feces, mainly consisting of HSYA degradation products stripped of their sugar groups or other fragments, primarily excreted in feces (Jin-Feng et al., 2014). When HSYA was administered via intravenous injection, 10 metabolites were identified, with the primary metabolic pathways including dehydration, deglycosylation, glucuronidation, and methylation. Some metabolites underwent multiple steps of metabolism and were predominantly found in urine, a result that contrasts with the gavage administration study, suggesting a potential role of intestinal flora in the oral metabolism of HSYA (Wu et al., 2020). Xu’s investigation on the effects of HSYA on metabolic enzyme activities in rats showed that after 14 days of 3 and 12 mg/kg doses, HSYA significantly inhibited CYP1A2 activity and its mRNA expression while inducing CYP3A1 activity and its mRNA expression. Prolonged (14 days) or short-term (3 days) administration of a 12 mg/kg dose significantly inhibited CYP2C11 activity and its mRNA expression, with no significant effects on CYP2D4 activity. Given that HSYA may be used in combination with other drugs for cardiovascular diseases, understanding its interactions with metabolic enzymes is crucial for optimizing its clinical safety and efficacy (Li Z. et al., 2021). However, the full extent of HSYA metabolism remains poorly understood, and the key enzyme isoforms involved, along with their corresponding metabolic reactions and metabolites, require further elucidation.

Excretion: For intravenous administration, 88.6% of HSYA was excreted directly into the urine (Yang et al., 2009). For oral administration, the excretion of HSYA in feces exhibits the highest cumulative rate, followed by urine, whereas the excretion of HSYA in bile is the least in rats (Li, 2007). When rats were administered HSYA monomer, safflower extract, and compound cerebroside tablets, the highest excretion rate was found in the safflower extract group. The combination of compounds reduced HSYA excretion, suggesting that other constituents of safflower may affect HSYA absorption and utilization. Additionally, the compound combination may enhance the utilization of HSYA in safflower (Li C. Y. et al., 2015). A deeper discussion of the clinical pharmacokinetics of different administration routes is provided below.

Drug interaction: Some preclinical studies suggest that HSYA may enhance anti-tumor effects when combined with post-marketing cancer therapeutic agents. Cisplatin, which inhibits cancer cell proliferation by forming DNA adducts and inducing DNA damage, may have its oxidative damage reduced by HSYA, which regulates the cellular redox state. Moreover, HSYA may enhance the anti-tumor effect of cisplatin (Chen Y. et al., 2022). HSYA also increases cisplatin sensitivity in ovarian cancer cells by downregulating the PI3K/AKT pathway and enhancing DNA repair mechanisms, thus reversing cisplatin resistance (Shen et al., 2019). HSYA has also been shown to significantly improve 5-FU’s inhibitory effect on the proliferation and migration of colorectal cancer (CRC) cells, as well as to alleviate 5-FU chemoresistance (Wang et al., 2023). Although no data exist on the interaction of HSYA with other drugs in the context of SY injection, patients may experience mild adverse reactions such as fever, palpitations, cutaneous allergic papules, and somnolence (Guo et al., 2023).

### 4.1 Pharmacokinetics of oral administration

A clinical study on the pharmacokinetics of a single oral dose of HSYA in 12 healthy individuals showed that HSYA was rapidly absorbed, reaching peak concentration within 1 h, with an elimination half-life (t1/2) ranging from 2.6 to 3.5 h (Wen et al., 2008). This rapid elimination is attributed to the efficient breakdown of HSYA in the gastrointestinal tract and liver, facilitated by its high molecular weight and strong hydrophilicity (Wu et al., 2020). Therefore, improving the oral bioavailability of HSYA is essential for its effective clinical use.

HSYA is absorbed by passive diffusion, and the Caco-2 cell monolayer model demonstrates this transmembrane characteristic. To improve the permeability coefficient, several strategies have been proposed to develop novel drug delivery systems: (i) absorption enhancers, (ii) lipid complex formation, and (iii) P-glycoprotein (P-gp) inhibitors (Liang et al., 2012). Absorption enhancers, such as Chuanxiong volatile oil and polyethylene glycol 400, temporarily open intercellular tight junctions, facilitating passive diffusion of HSYA through cellular or bypass pathways (Liu et al., 2018). Lipid-based carriers, including microemulsions (Qi et al., 2011), solid lipid nanoparticles (Zhao et al., 2018), self-double-emulsifying drug delivery (Lv et al., 2012) and chitosan complex (Ma et al., 2015) have also been explored. As a P-gp substrate analog, HSYA’s absorption is affected by P-gp-mediated exocytosis, contributing to its low bioavailability. Consequently, P-gp inhibitors such as F-68 and Tween 80 may enhance HSYA’s oral absorption (Zhou et al., 2014). A suitable HSYA delivery system for clinical application is expected to be developed in the near future.

### 4.2 Pharmacokinetics of intravenous administration

Currently, SY injection, which contains HSYA, is administered intravenously in clinical practice. A pharmacokinetic study in 12 healthy Chinese female volunteers, following a single intravenous dose of SY injection (HSYA: 35, 70, 140 mg), revealed that HSYA was rapidly eliminated, following a two-compartment model with first-order elimination and a half-life of approximately 3 h (Yang et al., 2009). Li et al. studied 36 healthy Chinese volunteers of both sexes who received single-dose (25, 50, and 75 mg) and multiple-dose (50 mg daily for seven consecutive days) injectable HSYA lyophilized powder. The Cmax and AUC were dose-proportional and consistent with first-order kinetics. Notably, female subjects exhibited higher Cmax and AUC values than males, indicating the importance of considering gender differences in clinical dosing. Following multiple-dose administration (50 mg once daily for 7 days), HSYA was well tolerated by all subjects, with no accumulation observed (Li C. Y. et al., 2015). Moreover, combination therapy with HSYA and protocatechuic aldehyde significantly enhanced HSYA uptake (Zhao et al., 2020).

## 5 Safety

Regarding the embryotoxicity of HSYA, animal studies indicated that intravenous administration of HSYA (125, 250, and 500 mg/kg) to pregnant rats did not result in any significant changes in body weight, placenta weight, number of implantations, number of corpus luteums, number of live fetuses, or number of stillborn fetuses when compared to the control group. Therefore, HSYA is neither embryo- nor maternal toxic as verified by animal studies (Wang et al., 2016). In terms of drug metabolism, a group of 36 healthy Chinese volunteers received a single intravenous dose of pure HSYA injection, ranging from 25 to 75 mg. Blood concentration analysis revealed moderate linear pharmacokinetic (PK) characteristics across the dose range, suggesting that repeated administration does not result in drug accumulation. HSYA has a plasma protein binding rate of approximately 78%, indicating its prolonged effect (Wang et al., 2011) and does not competitively bind to other drugs. Consequently, HSYA demonstrates a high safety profile *in vivo* (Chu et al., 2006), though caution should be exercised when combining it with drugs that competitively inhibit HSYA plasma protein binding (Li C. Y. et al., 2015). In clinical applications of SY injection, which contains HSYA as the main component, most adverse drug events (ADEs) have been related to drug-induced allergic reactions (Liu et al., 2019), potentially due to antigenically active impurities present during the production process (Zheng, 2013).

## 6 Conclusion and prospect

The chemical compounds derived from TCM exhibit distinctive properties and significant efficacy in the prevention and treatment of various diseases. Numerous basic studies have demonstrated that HSYA possesses substantial potential as an anticancer agent, showing efficacy against a range of cancers, including liver, lung, gastric, colorectal, breast, ovarian and gliomas. This efficacy is likely attributable to the close association between HSYA and its ability to regulate several cellular processes, including cancer cell proliferation, invasion, metastasis, apoptosis, autophagy, and immune microenvironment modulation. Therefore, HSYA has substantial potential to inhibit cancer progression. A review of its molecular mechanisms reveals that HSYA’s anticancer effects offer the therapeutic advantage of being “multi-channel and multi-targeted.”

This review summarizes the physical and chemical properties, anticancer effects, pharmacokinetics, and safety of HSYA, with a focus on its anticancer mechanism. HSYA’s anticancer effects are mediated through the inhibition of transcription factors (NF-κB, β-catenin, ERK1/2, p38, TGF-β and mTOR), activation of transcription factors (G3K-3β and PPAR), promotion of mitochondrial autophagy, inhibition of autophagy flux, and regulation of apoptosis-related proteins (Bcl-2 and BAX). This leads to the release of cytochrome c, activation of cysteine proteases (caspases 3&9), and modulation of cell cycle drivers (cyclin D1) and EMT-associated proteins. Additionally, HSYA regulated the polarization of CD4^+^T cells within the tumor immune microenvironment, ultimately blocking cancer cell proliferation, transformation, and metastasis, leading to apoptosis. A comprehensive analysis of experimental studies from four databases showed that HSYA is a selective natural anticancer agent, both *in vitro* and *in vivo*. Cardamonin, a compound with a natural chalcone structure similar to that of HSYA, shares some molecular mechanisms with HSYA in exerting its anticancer effects. Cardamonin targets Bcl-2 family proteins, activating the caspase cascade, inhibiting the nuclear translocation of NF-κB, STAT3 and mTOR, blocks β-catenin signaling, downregulating cell cycle regulatory proteins, and inhibiting oncogenic signaling to induce apoptosis and arrest the cell cycle, thus halting proliferation and metastasis in various cancer cell types (Javaria et al., 2020). Among these, the NF-κB, PI3K/AKT/mTOR and Wnt-β-catenin pathways are also implicated in HSYA’s anticancer mechanism. However, ability of HSYA to regulate cancer progression via the JAK-STAT3 pathway has not been extensively studied. The JAK-STAT3 pathway is involved in cancer cell proliferation and survival (J et al., 2016), and recent studies have identified it as a novel target for cancer therapy (Gharibi et al., 2020; Zou et al., 2020). For example, cardamonin inhibits glioblastoma stem cell proliferation by suppressing the STAT3 pathway, which subsequently prevents the activation of Bcl-2, survivin, and VEGF (Ning et al., 2015). It also reduced STAT3 expression in gastric cancer cell lines by downregulating STAT3 phosphorylation, thereby reducing the expression of various carcinogenic proteins and inhibiting cancer cell growth (Zheng et al., 2019). Owing to the structural similarities between HSYA and cardamonin, it is plausible that the anticancer targets of HSYA may also include the JAK-STAT3 pathway (Gharibi et al., 2020).

Despite the promising anticancer properties of HSYA, its clinical applicability is limited by poor oral bioavailability due to low membrane permeability. To enhance the permeability of HSYA, various drug delivery systems have been developed, primarily focusing on lipid-based carriers. By reducing HSYA’s high water solubility, its bioavailability can be significantly improved. Additionally, microemulsions (Lopes, 2014), self-emulsifying systems (Lv et al., 2012), nanoparticles (Zhao et al., 2018), and chitosan complex (Hong et al., 2017) have been explored as potential strategies to enhance HSYA’s bioavailability. Furthermore, studies have shown that co-administration of HSYA with other traditional Chinese medicines, such as Danggui-Honghua and Taoren-Honghua, can optimize HSYA’s absorption and efficacy (Pan et al., 2022). When comparing the pharmacokinetics of HSYA with those of Cendroxin Tablets, it was observed that other herbs in Cendroxin Tablets enhance HSYA absorption and increase its bioavailability (Feng et al., 2012). Notably, HSYA administered via the gastrointestinal tract in healthy rats exhibited the highest plasma concentrations but was not detectable in the brain (Chu et al., 2006). However, injectable HSYA can easily penetrate a damaged blood-brain barrier (BBB) (Liang et al., 2018). HSYA injection has been approved for the treatment of stroke, a condition in which the BBB is significantly impaired. This makes HSYA particularly effective in stroke treatment. However, its inability to cross the intact BBB limits its therapeutic potential for other brain diseases (Chen Q. et al., 2022), For instance, in the clinical treatment of glioma, all patients possess tumor regions with an intact BBB, which must be fully treated for effective outcomes (Arvanitis et al., 2020), Therefore, strategies to improve the BBB penetration of HSYA are essential. Some studies suggest that this challenge could be addressed by combining HSYA with drugs that have high BBB permeability but minimal systemic effects (Tang et al., 2021). Wu found that HSYA, in combination with aroma-opening drugs, promoted HSYA distribution in the rat brain, supporting the potential for developing brain-targeting drug delivery systems (Wang et al., 2014). While no preclinical studies have been conducted in this area, the drug has entered clinical trials and is marketed for neurological and cardiovascular diseases. In the case of acute ischemic stroke, Jilin TianSanQi Pharmaceutical Co., Ltd. has completed a phase 3 clinical trial (CTR20201900), and the drug has demonstrated favorable clinical outcomes. The study reached the primary endpoint, showing superior efficacy in patients with acute ischemic stroke, as evidenced by a higher proportion of subjects achieving an mRS score ≤1 after 90 days of treatment, with a favorable safety profile and no new safety concerns. In January 2023, Yuekang Pharmaceutical’s HSYA for injection also reached the primary endpoint in a phase III clinical trial in acute ischemic stroke patients, and the product is now in the process of applying for market approval in China (Acceptance No. CXZS2300027). While studies on the anticancer effects of HSYA are still in the early stages, further preclinical studies on its pharmacological mechanisms are necessary before advancing to clinical trials. Nonetheless, the promising efficacy and safety of HSYA in cardiovascular and cerebrovascular diseases offer valuable insights for its potential application in anticancer research.

Future investigations should explore a broader range of cancer types and molecular mechanisms related to HSYA. Additionally, studies should focus on improving its bioavailability, intestinal absorption, and *in vivo* stability within relevant cancer models, providing valuable information to support its future clinical application.
